# Estrogen-Dependent Changes in Dura Mater Microvasculature Add New Insights to the Pathogenesis of Headache

**DOI:** 10.3389/fneur.2017.00549

**Published:** 2017-10-18

**Authors:** Olga V. Glinskii, Virginia H. Huxley, Vladislav V. Glinsky

**Affiliations:** ^1^Research Service, Harry S. Truman Memorial Veterans Hospital, Columbia, MO, United States; ^2^Department of Medical Pharmacology and Physiology, University of Missouri, Columbia, MO, United States; ^3^Center for Gender Physiology and Environmental Adaptation, University of Missouri, Columbia, MO, United States; ^4^Department of Pathology and Anatomical Sciences, University of Missouri, Columbia, MO, United States

**Keywords:** headache, estrogen, dura mater, microvasculature, nociceptive neurons

## Abstract

The pathogenesis of headaches is a matter of ongoing discussion of two major theories describing it either as a vascular phenomenon resulting from vasodilation or primarily as a neurogenic process accompanied by secondary vasodilation associated with sterile neurogenic inflammation. While summarizing current views on neurogenic and vascular origins of headache, this mini review adds new insights regarding how smooth muscle-free microvascular networks, discovered within dura mater connective tissue stroma (previously thought to be “avascular”), may become a site of initial insult generating the background for the development of headache. Deficiencies in estrogen-dependent control of microvascular integrity leading to plasma protein extravasation, potential activation of perivascular and connective tissue stroma nociceptive neurons, and triggering of inflammatory responses are described. Finally, possible avenues for controlling and preventing these pathophysiological changes are discussed.

## Introduction

The impact of headache, including migraine, on society is considerable both in terms of reduced quality of life and financial burden. There are two main hypotheses of the pathogenesis and the origin of headaches: vasogenic (1, 2) and neurogenic (3–5). According to the vasogenic hypothesis, headache originates from vasodilation and activation of nociceptors innervating meningeal blood vessels, followed by vasoactive neuropeptide release and onset of pro-inflammatory reactions. The neurogenic hypothesis states that CNS disorders, such as cortical spread depression lead to nociceptive afferent activation causing alteration in intracranial vessel diameter and blood flow. These changes serve as a trigger of the trigeminovascular system followed by vasoactive neuropeptide release [particularly calcitonin gene-related peptide (CGRP)] and pro-inflammatory reaction. Importantly though, recent data show that different cellular microenvironments and distinct vessel wall anatomical features in meninges and cortex can elicit opposing vascular effects in response to the very same treatment (6).

**Figure 1:**
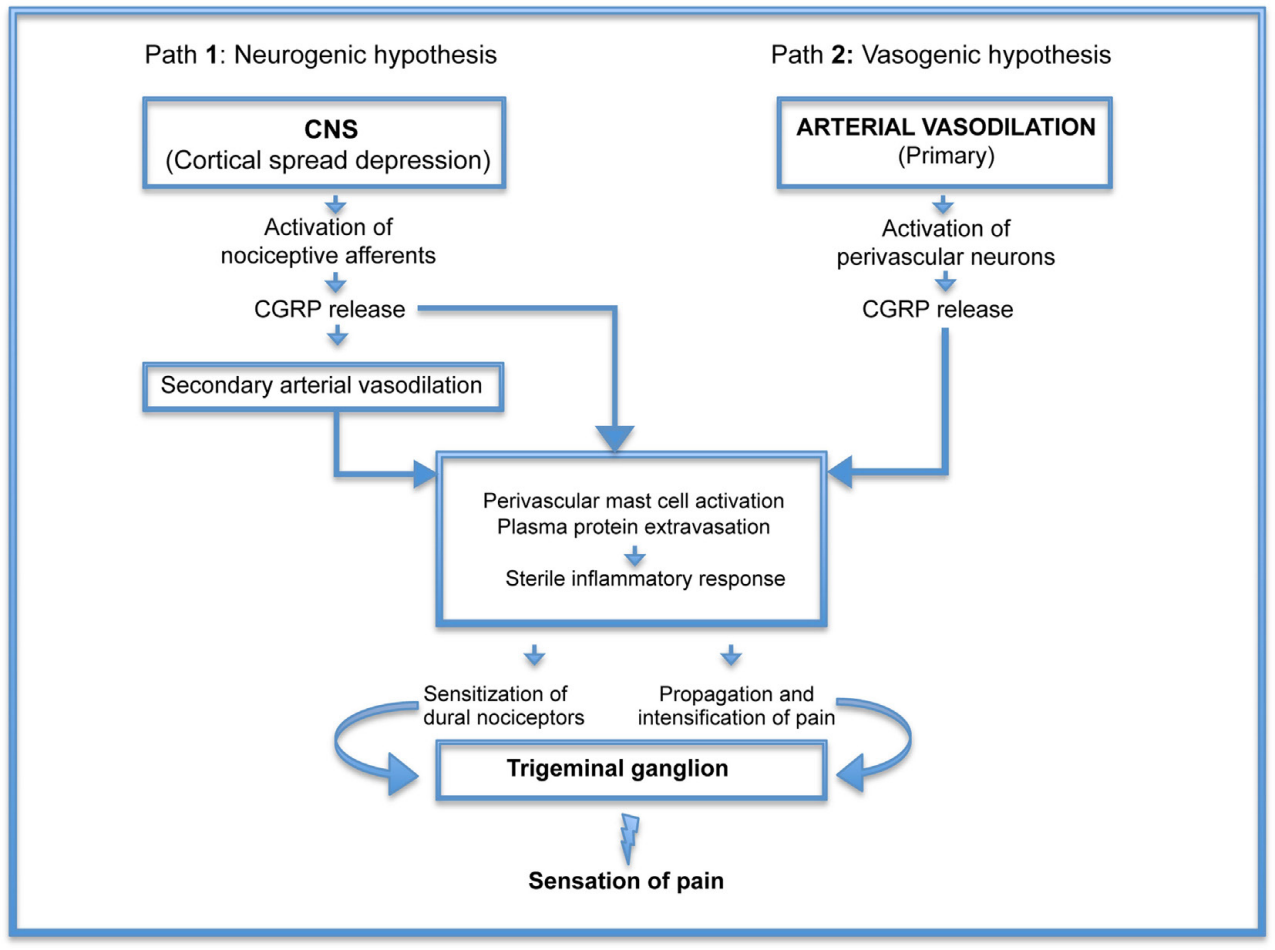
Pathogenesis of headache: schematic representation of neurogenic (Path 1) and vasogenic (Path 2) hypotheses demonstrating that the real difference between the two is the early mechanisms leading to calcitonin gene-related peptide (CGRP) release. Once the process is initiated, the pathogenesis of headache appears to follow the same route.

Figure 1, illustrating these hypotheses, demonstrates that the real difference between vasogenic and neurogenic hypotheses focuses on the early mechanisms leading to CGRP release. Once the process is initiated, the pathogenesis of headache appears to follow the same route. Thus, elucidation of the initiating factors could be beneficial not only for the treatment of headache but also, more importantly, for the prevention of this illness.

## Headaches in Women Associated with Estrogen “Withdrawal”

Despite the multifactorial causes of headache, two widely accepted risk factors for this disorder are female gender and sex hormone status (7–9). By the third decade of life, incidence of headache, including migraine, is threefold higher in women than men (3), with estrogen (E2) reduction being the main trigger for this pathological condition. Estrogen reduction, or “withdrawal,” occurs at various stages of a woman’s life: during the late luteal phase of the natural menstrual cycle (10, 11), during the hormone-free interval of combined oral contraceptives (12), following surgical menopause, following delivery, and during peri- and post-menopausal periods. Any of these stages may link to the development of headache (10–12). Mechanistically, estrogen-driven changes resemble an inflammatory response during the menstrual cycle (13) with significantly increased inflammatory cytokine production following ovulation (mostly from monocytes and/or macrophages) when E2 levels are low (14, 15). The link of reduced E2 with initiation of an inflammatory response is not limited to the vasculature as a decline in E2 levels has been shown to increase pro-inflammatory reactions within bone (16). Although the precise mechanisms of headache associated with reduced E2 levels are still unknown the proposed theory that the inflammation within the dura mater, resulting in pain, is fundamental to the pathogenesis of this disorder (17). The question is: what changes within cranial dura mater associated with estrogen deprivation trigger pro-inflammatory reactions in the tissue that contribute to symptoms of clinically occurring headache.

## Anatomical Features of Cranial Dura Mater are Decisive for the Pathogenesis of Estrogen Deprivation-Mediated Headache

Similar to the brain, cranial dura mater is situated inside the intracranial cavity, an indistensible closed space uniquely sensitive to changes in pressure, and a chemically privileged site (18). As Strassman and Levy (18) point out, “The distinctive clinical characteristics of headache may prove to be related not so much to any differences in the intrinsic molecular or cellular properties of the meningeal sensory neurons but rather to the distinctive properties of the tissue that they innervate,” which is consistent with recent findings showing that the very same treatment with the migraine trigger glyceryl trinitrate can simultaneously induce opposite reactions: vasoconstriction in meningeal and vasodylation in cortical microvasculature (6). Thus, different physiological or pathological processes within the intracranial dura, most heavily innervated by nociceptive afferents, will certainly affect meningeal sensory neuron activity.

Another distinctive feature of the intracranial dura is its high population of immune cells including macrophages, dendritic cells, and mast cells (19, 20). This combination of cell types provides the morphological prerequisites for delayed sterile inflammation (21–23). Mast cells can become activated in response to a range of physiological stimuli, including estrogen fluctuations. Interestingly, dural mast cells respond to estrogen deprivation by switching their phenotype (24). It was shown that female rats during diestrus had the highest density of more mature phenotype of dural mast cells, suggesting that during estrogen “withdrawal” a trigger leading to mast cell activation could promote a more robust inflammatory reaction increasing nociceptive response (24).

The brain “capsule,” cranial dura mater, is the most pain sensitive structure. Both the sensory innervation as well as pain sensitivity are restricted to the meningeal covering around the outside of the brain and do neither extend into the brain itself nor its intrinsic blood vessels (18). Although dural sensory innervation is often referred to as a “vascular innervation,” it is not specifically or even preferentially vascular as these nerves supply both vascular and non-vascular dural territories (18, 25). Further, there is an enrichment of CGRP content in meningeal sensory neurons compared with trigeminal ganglion cells innervating extracranial skin (26). The majority of neurons project to mechanosensitive receptive fields on the dura, from which they could be activated by mechanical stimuli. Stimulation of dural receptive fields during local connective tissue fluid accumulation, as a result of compromised vessel wall integrity, may activate mast cells and directly excite the neurons and/or enhance their mechanical sensitivity (27), so that they can be activated strongly by mechanical stimuli that otherwise would have evoked little or no response. One of the most important mechanisms whereby intradural mechanosensitivity can be changed is if the fluid balance across the vasculature is shifted toward the interstitial fluid accumulation, which can lead to the inflammatory state (28). Thus, any circumstances leading to compromised vessel wall integrity associated with plasma protein leakage and extravasation of leukocytes will result in local tissue swelling, mast cell activation (29), augmented dural mechanosensitivity (29, 30), and, without resolution (which requires microvascular integrity), delayed inflammatory reaction. The combination of increased dural mechanosensitivity and a delayed inflammatory response is likely to create the background for headache.

## Estrogen “Withdrawal” Initiates Vascular Remodeling and Dural Micro-Environmental Changes with Unique Functional Outcome Forging the Background for Estrogen-Dependent Types of Headache

Data from the studies on dura mater in ovariectomized pigs (31) suggest that the primary headache following estrogen perturbation, particularly during estrogen “withdrawal,” is a consequence of structural changes within cranial dura related to compromised microvessel integrity. To analyze vascular integrity, a perfused dura mater model was observed using high-resolution digital epifluorescence, laser scanning confocal microscopy and computer-assisted morphometric analysis (31). The results of the advanced microscopic analysis revealed that large areas of the connective tissue stroma, thought previously to be avascular, possessed extensive microvascular networks (32–37). Further, the cessation of the ovarian hormone production following bilateral ovariectomy in Yucatan miniature pigs caused gradual (within 2 months) yet significant remodeling of these microvascular networks. This remodeling was characterized by a threefold decrease in microvessel density, capillary rarefaction, and increase in average microvessel diameter (31). Moreover, augmented fluorescent lectin accumulation in the perivascular space immediately adjacent to the capillaries and small venules indicated that this process altered the functional properties of terminal microvessels related to permeability and water–solute exchange. The increase in perivascular marker accumulation was almost fourfold in ovariectomized pigs compared with intact females (31).

The increase in microvascular leakage within cranial dura mater suggests that this process could be a trigger of a dural inflammatory response and initiation of headache. Indeed, meningeal edema was observed using single photon emission computed tomography in a preliminary study in humans during migraine attacks (38). In fact, dural inflammation with local tissue edema is thought to not only lower the nociceptive threshold of dural afferents facilitating nociceptive transmission to the central nervous system (18) but also enhance intracranial mechanosensitivity (39). Activation of perivascular and stromal mechanoreceptors, an important part of defense mechanism in the cranial dura contained within an essentially closed space, is widely accepted in the pathogenesis of headache. Mechanistically, alterations mediated by estrogen “withdrawal” in cranial dura were shown to result from disrupted estrogen-dependent control of angiopoietin-1/Tie-2 (21) and PDGF-BB signaling (40) involving both vascular endothelial cells and pericytes. Disturbed signaling from both molecular pathways may contribute to the marked increase in microvessel permeability following estrogen deprivation as estrogen regulation of endothelial/pericyte interactions is essential in stabilizing meningeal microvessels and maintaining healthy microvascular networks (40). Detailed analysis of dural vascular leakage in ovariectomized pigs (31) suggests associated connective tissue swelling and local changes resulting in mechanosensitive neuron and mast cell activation with a subsequent inflammatory response. However, a major question still remains: where is the primary origin of mechanosensitive neuron activation during estrogen “withdrawal”?

## “Which Came First? The Chicken or the Egg?”

As part of the pathophysiological mechanisms of headache, the trigeminovascular system is activated (putatively as a defense mechanism) initiating neuropeptide release and activation of the trigeminal nucleus caudalis with subsequent mediation of pain (41, 42). This pain is characterized by features consistent with exaggerated intracranial mechanosensitivity (39). Adequate stimulus from meningeal trigeminal nociceptors (arising from neurogenic or/and vascular components) triggers the activation of the dural nociceptive system, which is an alarm system indicating the presence of endogenous or exogenous noxious factors affecting the CNS. The CNS, in turn, elicits a pain response. However, the question whether a primary insult has neurogenic or vascular origin remains open. Consideration of the anatomical arrangement of the cranial dura mater provides evidence that inputs from both vascular and non-vascular (stromal) sensory stimulations are integral to the control of trigeminal nuclei complex activation. The majority of sensory neurons that secrete neuropeptides, including CGRP, terminate as free (43), encapsulated and lamellated nerve terminals (21) in two main locations. The first, accounting for ~20%, is at segments of meningeal vessels coursing alongside the arteries and venous sinuses (where sensory neuron activation or pain could be evoked by vasodilation as a consequence of vascular smooth muscle relaxation, supporting the vascular theory). The second, ~80%, is in the dura connective tissue proper (43) where smaller bundles or single fibers branch off and take a vessel-independent course (44, 45). There are two types of nerve terminals exposed to the collagenous fiber system: Ruffini-like terminals (41) slowly adapting mechanoreceptors detecting intracranial tensile forces (15) and rapidly adapting (lamellated) receptors (46) responding to volume changes. Those peptidergic nerve fibers have great functional significance in the context of neurogenic inflammation. It has been suggested that trigeminal sensory fiber activation leading to neurogenic inflammation with neuropeptide release, vasodilation, plasma protein extravasation, and mast cell degranulation is an important part of the pathogenesis of migraine (47). However, plasma extravasation, the major component of the inflammatory response, may develop by neurogenic or non-neurogenic mechanisms. Plasma extravasation can be induced by local release of substances, such as serotonin and histamine (48, 49) from activated mast cells (in response to dural tissue mechanoreceptors), and these compounds alter blood vessel permeability directly. The other mechanisms compromising vessel wall integrity revealed during estrogen “withdrawal” (31) lead to vessel leakage at the sites of vasculature void of smooth muscle cells (capillaries and venules) and, therefore, lacking vasodilation. Of note, endothelium of postcapillary venules is a target for the mediators of both neurogenic and non-neurogenic plasma extravasation (50–52). Under certain conditions, the endothelium appears to trigger a cascade of biochemical and physiological events, which can lead to the formation of interendothelial cell gaps and leakage of macromolecules (52). As Miller and Sims summarize, “These events might include calcium entry, formation of prostaglandins and lipoxygenase products, and generation of free radicals (53). In as much as protein leakage occurs within the venules (50, 51), it is unlikely that vascular smooth muscle is involved in any direct way.” Thus, the pathophysiological concept of vascular headaches, based on the notion that changes in vessel diameter would trigger pain, should be modified to include consideration of changes in microvascular wall integrity independent of resistance vessel vasodilatation. While the issue of vascular versus neurogenic mechanisms of origin in primary headaches such as migraine and cluster headache remains unresolved, recent data suggest that the smooth muscle-free part of the microvasculature (capillaries and small venules) located in what had been thought previously to be “avascular” connective tissue regions, could serve as the primary sites of trigeminovascular system activation in response to estrogen deprivation (40).

## Potential Role for the Microvasculature in Mediating Estrogen “Withdrawal” Headaches: New Data

Recent progress has contributed to better understanding of the mechanisms by which estrogen “withdrawal”-mediated structural changes within dura mater tissue lead to functional outcome creating the background for the headaches. The novel insight is that the microvasculature may be the site of initial insult in headache. The contribution of the microvasculature may well have been overlooked given the difficulty of observing the rich network of capillaries and venules coursing in among the 80% of the apparently “non-vascular fibers.” Reduction of estrogen causes local disruption of the intimate association between pericytes and endothelial cells in capillaries and venules (31, 40). This results in a direct alteration of the barrier properties leading to increased fluid and solute flux independent of changes in arteriolar feed vessel diameter. Fluid flux from the microvessels in turn leads to an elevation in interstitial fluid pressure and swelling of matrix glycoproteins. Dural fibrous structure contains proteoglycans and glycoproteins. Due to their glycosaminoglycan (GAG) chains, these molecules concentrate negative charge and are highly hydrophobic. This combination results in the attraction of ions while repelling fluid thereby creating an osmotic imbalance that results in the GAG absorbing water from surrounding areas and tissue swelling (54). As all of this occurs in the enclosed intracranial space containing the brain; the resulting local edema impinges on the neurons coursing through that area initiating the mechanosensitive pain response. Recent data show that non-arterial diffuse dural innervation, contains a majority of peptidergic fibers including free nerve endings of CGRP-expressing C fibers. This innervation is far more extensive than the arterial innervation and is distributed among a pervasive capillary network within previously thought to be “avascular” connective tissue stroma (55). Compromised capillary barrier properties associated with tissue swelling results in parenchymal cells activation, including macrophages and mast cells, further amplifying the inflammatory response. Physiological or pathological events occurring within and between vascular cells could mediate bidirectional communication between meningeal nerve fibers (sensory and sympathetic) and cells comprising the associated blood vessels without the need for changes in vascular tone. Therefore, afferent input from cranial dura mater can influence or be impacted by blood vessels (56). Accumulation of extravasated material due to compromised small vessel integrity may lead to infiltration of immune cells into nearby tissue and release of cytokines known to sensitize sensory afferents (30). Thus, microvessel integrity-dependent activation of non-arterial diffuse innervation (with vast CGRP-containing fibers) would be a more likely source of CGRP-mediated tissue inflammatory response than the lesser innervation of meningeal arterioles, possessing a smooth muscle cell component (55). Finally, most of the responses are “self limiting” in that increases in tissue pressure will limit additional fluid flux; should vasodilatation occur upstream, the subsequent elevation in microvessel hydrostatic pressure will likely be offset by the elevated extravascular pressure (57). Resolution of headache would be hypothesized to include not only removal of the excess fluid but also restoration of the microvascular endothelial/pericyte interactions and microvascular barrier integrity.

## Can E2 Treatment Protect Estrogen Deprivation-Induced Microvascular Damage?

**Figure 2:**
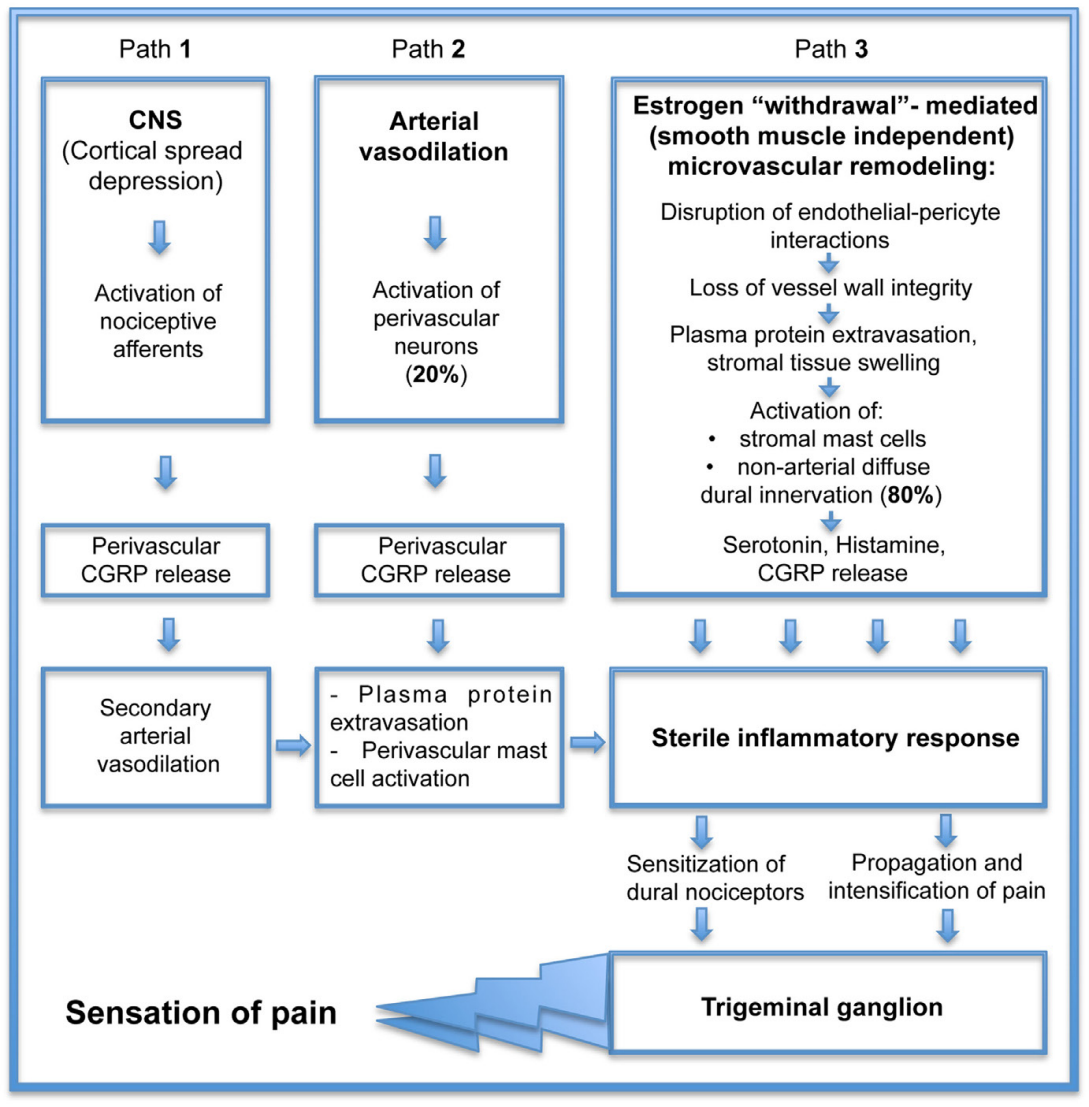
Consequential contribution of small vessel remodeling in the pathogenesis of estrogen “withdrawal”-mediated headache.

If estrogen withdrawal precipitates headache attack, estrogen supplementation should prevent it. In cell culture and animal models, estrogen administration has been shown to be anti-inflammatory and vasculoprotective (14, 58). Unfortunately, estrogen can also produce pro-inflammatory and vasotoxic effects in women whose hormone levels are reduced (7, 59). Clinical studies have raised many important questions about the vascular effects of estrogen that, as yet, cannot easily be answered. Does this mean that estrogen cannot be used to reduce headache? In fact, it appears to not be a “yes/no” answer. One hint comes from studies comparing a flat dose hormone replacement therapy (FD HRT) designed to maintain diestrus estrogen levels to a pulsed dose replacement therapy (P HRT) mimicking physiological E2 fluctuations associated with an estrous cycle (28 days) in pigs following ovariectomy. The simple constant low dose E2 treatment in FD HRT demonstrated a vascular network similar to the abnormal network found during E2 deprivation following ovariectomy (40). The vasculature displayed capillary degradation and vascular leakage within non-arterial microvascular networks. Intriguingly, vascular integrity was maintained by an E2 pulse treatment (40), suggesting that protection of vessel integrity by P HRT can eliminate the sequential processes of microvascular leakage with connective tissue swelling, tissue macrophage and mast cell activation, and ultimately prevent inflammatory mediators release (associated with those sequential events). Currently, the pathogenesis of primary headaches remains obscure and a matter of ongoing discussion of the vascular phenomenon that resulted from intracranial vasoconstriction followed by rebound vasodilation (Figure 2, path 2) and the neurovascular theory (Figure 2, path 1), describing headache as primarily a neurogenic process with secondary changes in vascular perfusion associated with a sterile neurogenic inflammation. Although basic and clinical research continue to offer complementary approaches that are improving our understanding of the role of CGRP in the complex pathogenesis of headache, the major part of this pathogenesis related to the microvasculature within dura connective tissue stroma (Figure 2, path 3) is missing. Estrogen “withdrawal”-mediated changes in cranial dura mater reveal the additional, smooth muscle-independent mechanisms involving microvascular alterations within large, previously thought to be “avascular” areas with non-arterial diffuse dural innervation, distributed across a dense capillary networks with 80% of peptidergic CGRP-expressing fibers (55). Those microvascular changes, evoked by estrogen deprivation, lead to plasma protein extravasation and stromal tissue swelling, initiating the mechanosensitive pain response with subsequent macrophage and mast cell activation further supporting sterile inflammatory response with inflammatory cytokines release, resulting in sensitization of dural nociceptors, trigeminal ganglion activation, and the ultimate sensation of pain. If P HRT can eliminate estrogen “withdrawal”-mediated deleterious microvascular changes, it should prevent the development of a tissue inflammatory reaction. While such an approach is promising, further studies are required to elucidate the effect of P HRT on local inflammatory cytokine production, the key element in activation and sensitization of meningeal nociceptors (30).

## Author Contributions

Conception and design of the work; acquisition, analysis, and interpretation of data; drafting the work and revising it critically for important intellectual content; final approval of the version to be published: OG, VH, and VG.

## Conflict of Interest Statement

The authors declared no potential conflicts of interest with respect to the research, authorship, and/or publication of this article.
